# Fine Particulate Matter Air Pollution and Mortality Risk Among US Cancer Patients and Survivors

**DOI:** 10.1093/jncics/pkab001

**Published:** 2021-02-21

**Authors:** Nathan C Coleman, Majid Ezzati, Julian D Marshall, Allen L Robinson, Richard T Burnett, C Arden Pope

**Affiliations:** Department of Economics, Brigham Young University, Provo, UT, USA; MRC Centre for Environment and Health, School of Public Health, Imperial College London, London, UK; Department of Civil and Environmental Engineering, University of Washington, Seattle, WA, USA; Engineering and Public Policy, Carnegie Mellon University, Pittsburgh, PA, USA; Health Canada, Ottawa, Ontario, Canada; Department of Economics, Brigham Young University, Provo, UT, USA

## Abstract

**Background:**

Exposure to fine particulate matter (PM_2.5_) air pollution has been linked to increased risk of mortality, especially cardiopulmonary and lung cancer mortality. It is unknown if cancer patients and survivors are especially vulnerable to PM_2.5_ air pollution exposure. This study evaluates PM_2.5_ exposure and risk for cancer and cardiopulmonary mortality in cohorts of US cancer patients and survivors.

**Methods:**

A primary cohort of 5 591 168 of cancer patients and a 5-year survivor cohort of 2 318 068 was constructed using Surveillance, Epidemiology, and End Results Program data from 2000 to 2016, linked with county-level estimates of long-term average concentrations of PM_2.5_. Cox proportional hazards models were used to estimate PM_2.5_-mortality hazard ratios controlling for age-sex-race combinations and individual and county-level covariables.

**Results:**

Of those who died, 26% died of noncancer causes, mostly from cardiopulmonary disease. Minimal PM_2.5_-mortality associations were observed for all-cause mortality (hazard ratio [HR] = 1.01, 95% confidence interval [CI] = 1.00 to 1.03) per 10 µg/m^3^ increase in PM_2.5._ Substantial adverse PM_2.5_-mortality associations were observed for cardiovascular (HR = 1.32, 95% CI = 1.26 to 1.39), chronic obstructive pulmonary disease (HR = 1.10, 95% CI = 1.01 to 1.20), influenza and pneumonia (HR = 1.55, 95% CI = 1.33 to 1.80), and cardiopulmonary mortality combined (HR = 1.25, 95% CI = 1.21 to 1.30). PM_2.5_-cardiopulmonary mortality hazard ratio was higher for cancer patients who received chemotherapy or radiation treatments.

**Conclusions:**

Air pollution is adversely associated with cardiopulmonary mortality for cancer patients and survivors, especially those who received chemotherapy or radiation treatment. Given ubiquitous and involuntary air pollution exposures and large numbers of cancer patients and survivors, these results are of substantial clinical and public health importance.

Epidemiological and related research provides substantial evidence that fine particulate matter (PM_2.5_, particles ≤ 2.5 µm in aerodynamic diameter) air pollution contributes to increased risk of cardiovascular and respiratory disease (1,2). PM_2.5_ air pollution contributes to global burden of disease and to premature mortality (3‐6). Cohort studies have identified associations between long-term exposure to PM_2.5_ and all-cause, cardiopulmonary, and lung cancer mortality (7‐14). Several studies have also found limited evidence of an association between mortality or incidence from various nonlung cancers and air pollution (15‐21).

PM_2.5_-mortality associations have been primarily observed in broad populations or in population-based cohorts. It remains unclear if there are specific, identifiable subpopulations that are more vulnerable or susceptible to long-term air pollution exposure. For example, a recent study observed relatively large PM_2.5_-mortality associations in a cohort of cardiac transplant patients (22). Adverse PM_2.5_-mortality associations have also been observed in specific cohorts of cystic fibrosis (23), myocardial infarction (24), and tuberculosis (25) patients and survivors.

Cancer patients and survivors may be especially vulnerable to exposure to PM_2.5_ air pollution. Cancer treatments, including chemotherapy and radiation therapy, can have adverse effects on the cardiovascular and respiratory systems (26,27). It is unknown if exposure to air pollution contributes additional cancer mortality risk (28) or further contributes to cardiovascular or respiratory mortality risk in cancer patients or survivors. The primary objective of this study is to evaluate associations between long-term PM_2.5_ exposure and mortality risk in large cohorts of US cancer patients and survivors, constructed from publicly available cancer registry data. Secondary objectives include 1) evaluate PM_2.5_-mortality associations in a cohort of 5-year survivors, 2) evaluate PM_2.5_-mortality associations using a time-dependent model with various lag structures, 3) evaluate PM_2.5_-mortality risks across subgroups with different types of cancer and for cancer types with different levels of survivability, 4) explore PM_2.5_-mortality associations specifically for patients treated with chemotherapy or radiation therapy, and 5) explore result sensitivity to modeling assumptions.

## Methods

### Study Subjects

**Figure 1.**
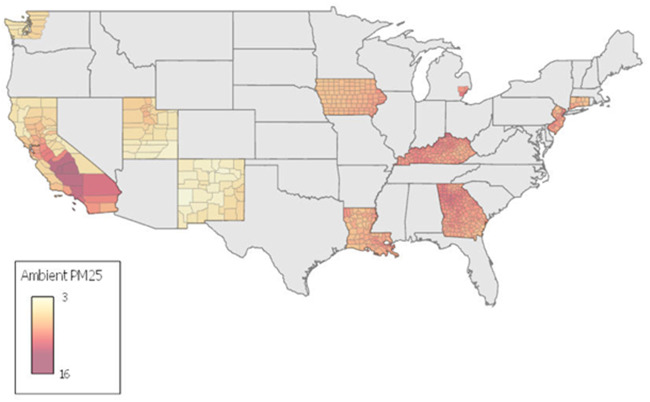
Estimated population weighted mean (1988-2015) PM_2.5_ concentrations (µg/m^3^) of US counties in the US National Cancer Institutes’ database.

Cancer incidence data were obtained from the US National Cancer Institute’s Surveillance, Epidemiology, and End Results (SEER) Program (29). SEER data are publicly accessible but require a signed SEER research data agreement (30). SEER data contain individual-level cancer incidence from 1975 to 2016 (31) collected from cancer registries across the United States. Registry locations are illustrated in Figure 1. Data regarding age, marital status, treatment status, mortality status, cause of death, survival time (month diagnosed with cancer to month of mortality or end of follow-up), SEER registry, and county of residence at the time of diagnosis were available.

Two cohorts were constructed using individuals aged 0-85 years with complete data. The first incidence of cancer was treated as the initial diagnosed cancer for individuals who had more than 1 cancer incidence. Individual cancer patients and survivors were followed up until date of death, until they were lost to follow-up, or until end of follow-up period (December 31, 2016). The primary cohort used in this analysis included 5 591 168 patients and 2 273 354 deaths, constructed from SEER cancer cases from 2000 to 2016. A 5-year survivor cohort consisted of 2 318 068 SEER cases and 457 463 deaths that survived 5 or more years.

Mortality information (based on the National Death Index), including cause of death, was collected by SEER. All-cause mortality was defined as all deaths not including accidents and adverse effects, homicide, or suicide. Cardiopulmonary mortality was classified as death from cardiovascular disease (International Classification of Diseases (ICD)-10 codes I00-I09, I11, I13, I20-I51), cerebrovascular disease (I60-I69), chronic obstructive pulmonary disease (COPD) (J40-J47), or pneumonia and influenza (J09-J18). Cancer deaths were classified as C00-C97. ICD 10 codes for specific cancer types are provided in Supplementary Table 1 (available online). Deaths not classified as cardiopulmonary or cancer were classified as other.

### Air Pollution Exposure

Beginning in 1999, regulatory monitoring for PM_2.5_ was collected nationwide. County-level annual-average PM_2.5_ concentrations for 1999-2015 were estimated using an empirical model derived from these regulatory monitoring data within a universal kriging and partial least squares framework with hundreds of geographic variables, including land use, population, and satellite-derived measures of land use and air pollution. Hold-out cross-validation (CV) indicated good model performance (10-fold CV-R^2^ = 0.78-0.90). Documentation of this modeling approach is found elsewhere (13,32). Air pollution estimates are available at the Center for Air, Climate, and Energy Solutions website (https://www.caces.us/). Individuals were assigned average PM_2.5_ for the exposure window of 1999-2015 based on the county where they were initially diagnosed with cancer.

For use in sensitivity analysis, back-casted PM_2.5_ estimates for 1988-1998 were calculated by multiplying each county’s mean PM_2.5_ to PM_10_ ratio from 1999 to 2003 by the estimated PM_10_ for each year from 1988 to 1998 as described in detail elsewhere (13). In one sensitivity analysis, average concentrations for exposure window of 1988-2015 was assigned. Mean PM_2.5_ concentrations for these 2 windows of exposure (1999-2015 and 1988-2015) were highly correlated (*R* = 0.98, *P* < .001).

### Additional Covariates

In addition to individual-level information, SEER provides county-level demographic information (33) from 1990 to 2016 from the US Census and American Community Survey including educational attainment (percentage that did and did not graduate high school and percentage to have some college education); median (adjusted as 2017 dollars) income; home value; rent; and percent less than 150% poverty, unemployed, and working class. The percentage of individuals who did not have health insurance (available from 2008 to 2016) and were living in rural parts of the county (2010 estimates) was obtained from the census. Behavioral Risk Factor Surveillance System, National Health Interview Survey, and National Health and Nutrition Examination Survey data were obtained that provided county-level risk-factor information, including percent smoking (1997-2010) (34), percent alcohol consumption (2002-2012) (35), and percent physically active and obese (2001-2011) (36). These data were averaged over the period with available data and assigned at the county level. Finally, indicator variables for urban vs rural, region (Northeast, Midwest, South, Mountain West, Pacific West), and state were linked to individuals at the county level.

### Statistical Methods

PM_2.5_-mortality hazard ratios (HRs) and 95% confidence intervals (CIs) for all-cause, cancer, cardiovascular, cerebrovascular, COPD, pneumonia and influenza, cardiopulmonary combined, and other mortality associated with a 10 µg/m^3^ increase of PM_2.5_ were estimated using Cox proportional hazards models. Models were estimated using the PHREG procedure in SAS version 9.4 (SAS Institute, Cary, NC). For individuals who died during the follow-up period, survival times in months were provided by SEER. End of follow-up was date of death for those who died. Censored survival times, for survivors, were end of mortality follow-up (December 2016) or when they were lost to follow-up. For analysis of specific cause-of-death groupings, censored survival times for deceased were dates of death for any other cause of death. All models controlled for combinations of age (by 1 year), sex, and race (using the STRATA statement in the PHREG procedure). Additional individual indicator variables for marital status, urban vs rural, state, and cohort year were included in the models along with county-level variables that represented educational attainment, income, smoking, percent uninsured, alcohol consumption, physical activity, and obesity as both linear and quadratic terms.

To account for temporal variability in pollution exposure, and to evaluate the sensitivity of the proportional hazards assumption relative to its impact on the PM_2.5_-mortality hazard ratio estimates (37), a temporally decomposed analysis that allowed for time-varying exposure was performed as documented elsewhere (38). Briefly, the primary cohort was temporally decomposed into 17 yearly cohorts (2000-2016). An individual was included in a yearly cohort if he or she was diagnosed with cancer either within that year or in a previous year but had survived until at least January of that yearly cohort. Ages were updated for each yearly cohort. Survival times were calculated based on survival months in specific year of the cohort. To explore alternative exposures windows, 1-, 6-, and 12-year lagged moving average estimates of PM_2.5_ concentrations were assigned for every yearly cohort. The construction of yearly cohorts in this temporal decomposition analysis is illustrated in Supplementary Figure 1 (available online). Cox proportional hazards models were estimated for each year separately, and all coefficients were allowed to vary for each year, thus minimizing the potential of violating the proportional hazards assumption of the Cox model (37). The Kolmogorov-type supremum test for violation of the proportionality assumption was generally not statistically significant (*P* > .05 for 13 of the 17 yearly cohorts). After estimating the PM_2.5_-mortality hazard ratio for the 17 yearly cohorts, a random effects and fixed effect meta-analytic approach was used to estimate hazard ratios for the full study period. This temporally decomposed approach proved a sensitivity analysis on the proportionality assumption with respect to its impact on the estimated hazard ratios (37).

PM_2.5_-mortality risks were estimated separately for patients with cancer types and for combined cancer subgroups with high 5-year SEER (28) survival rates (≥85%, including breast, prostate, skin, other male, and endocrine cancers), low 5-year survival rates (≤35%, including lung, esophagus, stomach, liver, pancreas, other digestive, brain, and ill defined), and medium 5-year survival rates (including oral, small intestine, colon, rectal, nose, larynx, bone, soft tissue, other female, kidney, bladder, other urinary, other respiratory, and other nervous). Models were also estimated separately for subgroups of patients with any chemotherapy or radiation treatment (vs not or missing). Models were estimated for male vs female patients; White, Black, vs other race categories; different age groups; and cancer stage.

To explore model sensitivity to the inclusion of various covariates, the following alternative models were estimated: model 1, includes PM_2.5_ and controls for combinations of age, sex, and race by including them in the STRATA statement; model 2, adds indicator variables for state, cohort year, marital status, and urban vs rural; model 3, adds linear terms for educational attainment, income, smoking, uninsured, alcohol consumption, physical activity, and obesity; model 4 (the primary model), adds quadradic terms for the county-level covariables, including cancer stage as a covariate; model 5, same as model 4 but includes cancer stage in the STRATA statement.

Sensitivity of spatial control was evaluated by changing the spatial control variable from state to no spatial control, east vs west, region, or SEER registry. A model that included a state indicator variable but also clustered by county of residence was included. Several additional models were estimated to evaluate other aspects of the model including a model that used the longer 1988-2015 exposure window, a model that used age as the time axis (removed age at cancer incidence from the STRATA statement and changed survival time to age at death or age at end of follow-up), and a model that estimated the PM_2.5_-mortality associations using a Fine-Gray competing risks model to avoid bias from other causes of death.

## Results

**Figure 2.**
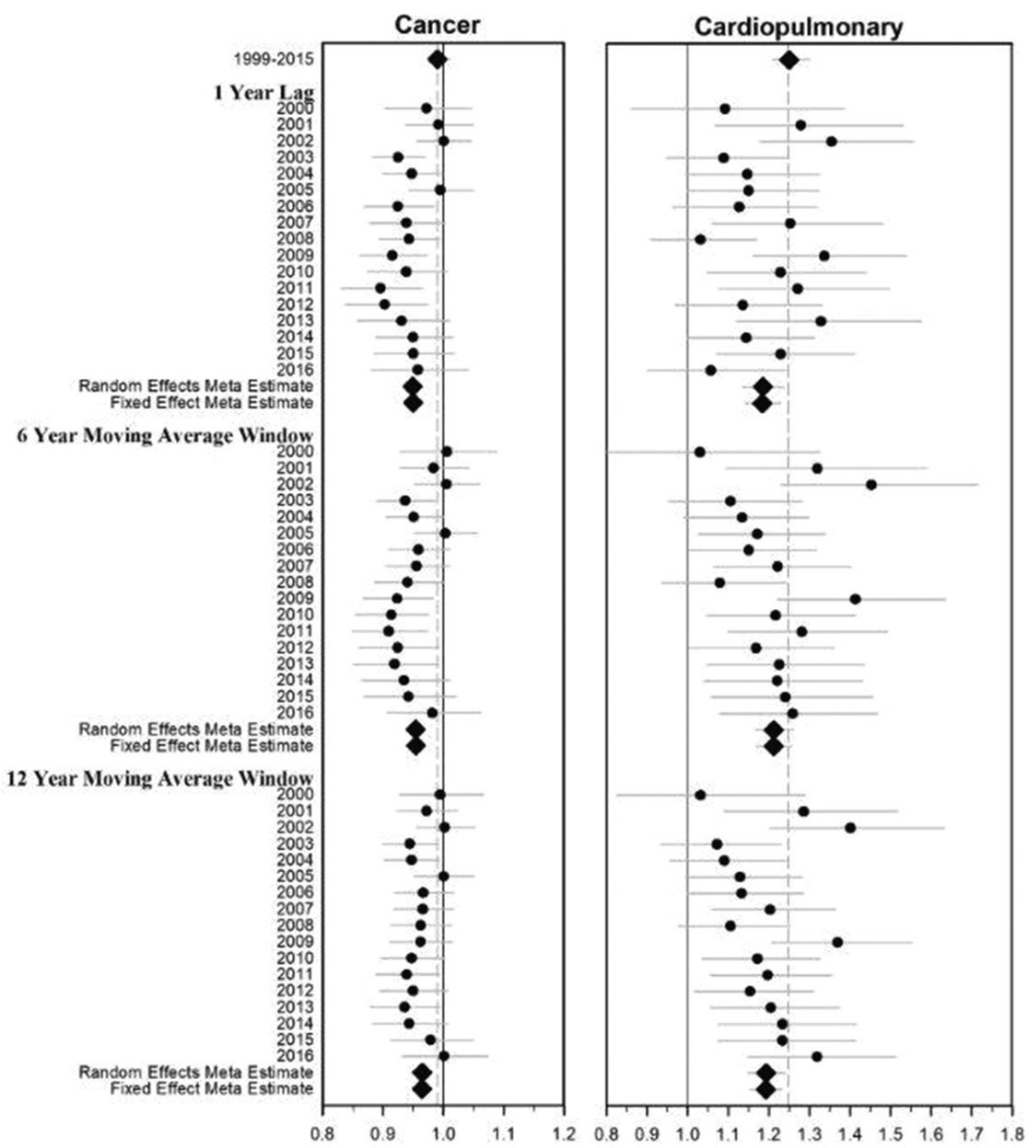
Estimated hazard ratios (95% confidence intervals) associated with 10 µg/m^3^ increase of PM_2.5_ and cancer and cardiopulmonary mortality using the primary cohort and various exposure windows for the temporally decomposed analysis.

Summary statistics for covariates included in the model are provided in Table 1. Table 2 presents the number of deaths and estimated PM_2.5_-mortality hazard ratios for the association between various causes of death and a 10 µg/m^3^ increase in PM_2.5_ for the primary and 5-year survivor cohorts. Of those who died, 25.7% died of noncancer causes, mostly from cardiovascular or respiratory disease. Cancer mortality was not associated with PM_2.5_. However, statistically significant PM_2.5_-mortality hazard ratios (per 10 µg/m^3^) were observed for cardiovascular (HR = 1.32, 95% CI = 1.26 to 1.39), COPD (HR = 1.10, 95% CI = 1.01 to 1.20), influenza and pneumonia (HR = 1.55, 95% CI = 1.33 to 1.80), and cardiopulmonary mortality combined (HR = 1.25, 95% CI = 1.21 to 1.30). Similar associations were observed using the time-dependent temporally decomposed analysis with the fixed effects meta estimate for cardiopulmonary mortality and 6-year lagged average of PM_2.5_ of 1.21 (95% CI = 1.17 to 1.26) (see Figure 2 or numeric estimates provided in Supplementary Table 2, available online).

**Table 1.** Summary statistics for the primary and the 5-year survivor cohorts.

Summary statistics	Primary cohort	5-yr survivor cohort
No. of individuals	5 591 168	2 318 068
Individual-level variables		
Age		
Average age, y (SD)	61.6 (14.6)	58.9 (14.7)
0-17 y, %	1.2	1.5
18-39 y, %	6.1	7.7
40-54 y, %	20.4	24.8
55-64 y, %	28.6	30.1
65-85 y, %	43.8	35.8
Sex		
Male, %	49.2	47.6
Race		
White, %	81.67	83.3
Black, %	11.2	9.9
Other, %	7.2	6.8
Marital status, %		
Divorced, %	9.0	7.9
Married, %	55.0	59.6
Partner, %	0.1	0.02
Separated, %	1.0	0.8
Single, %	15.6	14.5
Unknown, %	8.6	9.2
Widowed, %	10.7	8.0
Any chemo/radiation		
No or missing, %	55.5	59.3
Yes, %	44.5	40.7
Stage		
In situ (I) , %	6.9	10.6
Localized (II) , %	27.8	37.5
Regional (III) , %	15.8	14.8
Distant (IV) , %	17.2	5.3
Unknown or missing, %	32.3	31.7
County-level variables		
Population-weighted county PM_2.5_, µ/m^3^ (SD)		
1999-2015	11.2 (2.4)	11.2 (2.4)
1988-2015	13.2 (2.9)	13.2 (2.9)
Education		
No high school, % (SD)	19.3 (7.0)	18.9 (6.9)
Graduate high school, % (SD)	26.4 (6.5)	26.1 (6.4)
More than high school, % (SD)	54.4 (10.7)	55.0 (10.5)
Income		
Median income, US dollars (SD)	49 442 (12 332)	50 216 (12 381)
Median home value, US dollars (SD)	216 934 (117 002)	222 808 (117 696)
Median rent, US dollars (SD)	789 (209)	801 (207)
Poverty rate, % (SD)	22.1 (8.2)	21.7 (8.1)
Unemployed, % (SD)	7.4 (2.0)	7.4 (2.0)
Working class, % (SD)	63.3 (6.1)	62.9 (6.1)
Health		
Smokers, % (SD)	19.4 (5.0)	19.2 (4.9)
Consume alcohol, % (SD)	55.4 (10.2)	55.9 (10.1)
Obese, % (SD)	30.8 (4.9)	30.6 (4.9)
Physically active, % (SD)	76.3 (5.7)	76.5 (5.7)
Uninsured, % (SD)	15.3 (4.1)	15.2 (4.1)
Urban vs. rural		
Rural counties, %	8.7	7.7
Urban counties, %	91.3	92.3
Living in rural parts of county, % (SD)	14.0 (22.0)	13.0 (21.0)
State		
California, %	39.4	40.0
Connecticut, %	5.1	5.5
Georgia, %	11.7	11.1
Iowa, %	2.6	2.1
Kentucky, %	6.8	6.3
Louisiana, %	6.2	5.7
Michigan (Detroit) , %	5.5	5.7
New Jersey, %	13.2	13.9
New Mexico, %	2.2	2.2
Utah, %	2.4	2.7

Individual-level information is obtained at the time of diagnosis.

**Table 2.** Estimated HRs (95% CIs) by cause of death associated with 10 µg/m^3^ increase of PM_2.5_ exposure (average from 1999 to 2015) using the primary cohort and the 5-year survivor cohort^a^

Cause of death^b^	Primary cohort (n = 5 591 168)		5-year survivor cohort (n = 2 318 068)	
	No. of deaths	HR (95% CI)	No. of deaths	HR (95% CI)
All causes	2 273 354	1.01 (1.00-1.03)	457 463	1.00 (0.97-1.04)
All cancer	1 686 958	0.99 (0.98-1.01)	211 206	0.99 (0.95-1.04)
Cardiopulmonary	324 394	1.25 (1.21-1.30)	137 038	1.16 (1.09-1.23)
Cardiovascular	204 028	1.32 (1.26-1.39)	86 479	1.17 (1.09-1.26)
Cerebrovascular	41 734	1.08 (0.97-1.19)	18 281	1.11 (0.95-1.30)
COPD	59 328	1.10 (1.01-1.20)	24 027	1.07 (0.94-1.23)
Pneumonia/Influenza	19 304	1.55 (1.33-1.80)	8251	1.44 (1.15-1.81)
Other	262 002	0.87 (0.84-0.91)	109 219	0.84 (0.79-0.90)

a All models controlled for combinations of age, sex, and race and included subject-specific variables for marital status, state, urban or rural county, and year of cancer diagnosis. Models also included linear and quadratic terms for percentage in the county that smoke, consume alcohol, are physically active, are obese, are uninsured, live in rural areas, are below 150% poverty, unemployed, are working class, did not graduate high school, graduated high school, and did more school than high school, as well as median income, home value, and rent. CI = confidence interval; COPD = chronic obstructive pulmonary disease; HR = hazard ratio; PM_2.5_ = fine particulate matter.

b Cancer death is classified as C00-97; cardiopulmonary as I00-09, I11, I13, I20-51, I60-69, J40-47, J09-J18; cardiovascular as I00-09, I11, I13, I20-51; cerebrovascular as I60-69; COPD as J40-47; and pneumonia and influenza as J09-J18. Other was classified as all codes not cancer or cardiopulmonary.

**Figure 3.**
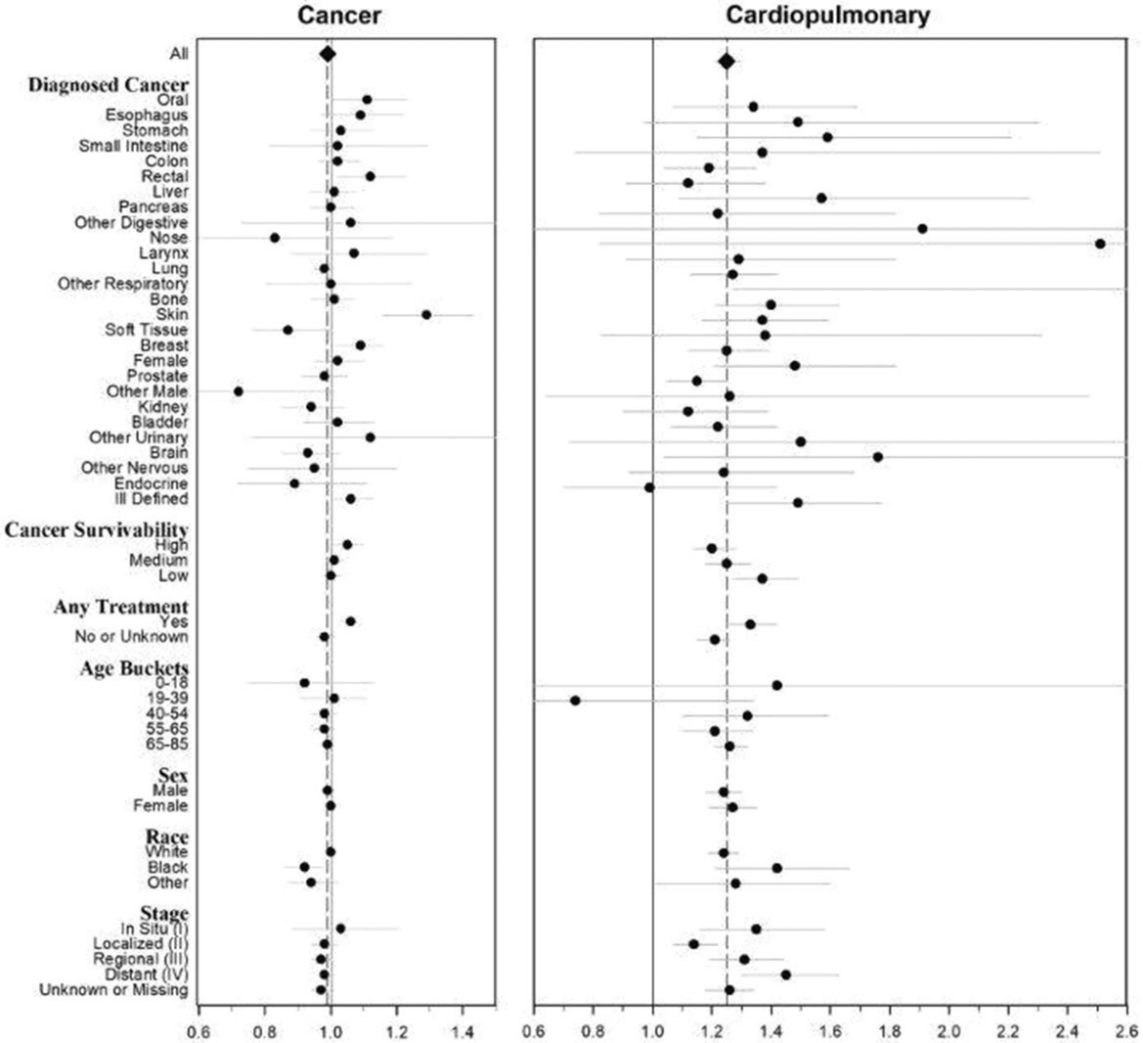
Estimated hazard ratios (95% confidence intervals) associated with 10 µg/m^3^ increase of PM_2.5_ and cancer and cardiopulmonary mortality stratified by selected subgroups using the primary cohort with 1999-2015 PM_2.5_.

Figure 3 illustrates results of analysis by subgroups of diagnosed cancer types, cancer-type groupings with different survival rates, treatment status, age, sex, race, and cancer stage from the primary cohort with PM_2.5_ exposure from 1999 to 2015 for cancer and cardiopulmonary mortality. Numeric results are provided in Supplementary Table 3 (available online). Statistically significant adverse PM_2.5_-mortality associations for cancer mortality were observed for oral and oropharyngeal, rectal, skin, breast, and ill-defined cancer types. Although no associations were observed between exposure to PM_2.5_ and cancer survival generally, there were associations for cancers with relatively high survival rates and for those individuals treated with chemotherapy or radiation. There was limited evidence of a differential effect of PM_2.5_ on cancer mortality across age, sex, race, and stage of cancer. PM_2.5_-cancer mortality associations were not highly sensitive to modeling choices (Supplementary Table 4, available online).

Adverse PM_2.5_-mortality hazard ratios for cardiopulmonary mortality were observed broadly across cancer subgroups. PM_2.5_-cardiopulmonary mortality hazard ratios were higher for cancer patients and survivors who received chemotherapy or radiation treatments vs those who did not, or treatment status was unknown. There was limited evidence of a differential effect of PM_2.5_ on cancer mortality for cancer survivability groups; age, sex, and race groups; or different stages of cancer. Results were not highly sensitive to modeling choices (Supplementary Table 4, available online).

## Discussion

Much evidence links PM_2.5_ air pollution with increased risk of cardiovascular and pulmonary disease mortality (1,2). Cancer patients and survivors have elevated risk of cardiovascular and pulmonary disease (26,27). It is unknown if exposure to air pollution further contributes to cardiopulmonary mortality in cancer patients and survivors. This analysis suggests that, after being diagnosed with cancer, extended exposure to PM_2.5_ air pollution may not increase the subsequent risk of dying of cancer generally. However, when grouping cancers by those with high, medium, and low 5-year survival rates, PM_2.5_-cancer mortality association were observed for cancers with relatively higher survival rates (HR = 1.05, 95% CI = 1.01 to 1.10) (Figure 2; Supplementary Table 3, available online). Adverse PM_2.5_-mortality associations were observed for several specific cancers including oral and oropharyngeal, rectal, skin, breast, and ill-defined cancers (Figure 3; Supplementary Table 3, available online), somewhat consistent with findings from other studies (15,17,21).

Statistically significant adverse PM_2.5_-mortality associations were observed with cardiovascular and respiratory mortality. PM_2.5_-mortality hazard ratios per 10 µg/m^3^ increase in PM_2.5_ were 1.32 (95% CI = 1.26 to 1.30) for cardiovascular mortality, 1.08 (95% CI = 0.97 to 1.19) for cerebrovascular mortality, 1.10 (95% CI = 1.01 to 1.20) for COPD mortality, 1.55 (95% CI = 1.33 to 1.80) for influenza and pneumonia mortality, and 1.25 (95% CI = 1.21 to 1.30) for cardiopulmonary mortality combined. These PM_2.5_-cardiopulmonary mortality associations are relatively large, suggesting that cancer patients and survivors are relatively sensitive to exposure to air pollution. For example, a recent meta-analysis of cohort studies of broad population-based cohorts estimated PM_2.5_-mortality hazard ratio for cardiopulmonary mortality per 10 µg/m^3^ increase in PM_2.5_ was 1.11 (95% CI = 1.08 to 1.14) (14), about half the excess risk observed in this cancer patient cohort. These results suggest that, after being diagnosed with cancer, extended exposure to PM_2.5_ air pollution is an important contributor to risk of dying of cardiopulmonary disease.

Another objective of this analysis was to explore potential differences in the PM_2.5_-mortality associations for cancer patients treated with chemotherapy or radiation therapy. The PM_2.5_-cardiopulmonary mortality hazard ratio was higher for cancer patients who were known to have received any chemotherapy or radiation (with or without surgery) treatments (HR = 1.33, 95% CI = 1.25 to 1.42; see Figure 3; Supplementary Table 3, available online). Chemotherapy or radiation may have adverse effects on the cardiovascular and respiratory systems, which may contribute to susceptibility to air pollution exposure. Unfortunately, chemotherapy and radiation data provided by SEER are limited in completeness and are influenced by other factors including patient preference, comorbidities, and proximity to treatment providers (39). A recent study of childhood cancer survivors observed that PM_2.5_ air pollution was more strongly associated with respiratory health-care encounters among chemotherapy-treated cancer survivors vs children without chemotherapy and a cancer-free sample (40).

The PM_2.5_-mortality association with pneumonia and influenza deaths in the cohorts of cancer patients and survivors was notably large, with a hazard ratio per 10 µg/m^3^ increase in PM_2.5_ of 1.55 (95% CI = 1.33 to 1.80). It is unknown if this relatively high hazard ratio for pneumonia and influenza deaths is partially due to the impact of cancer on the immune system or cardiorespiratory systems. A similarly large hazard ratio for the association between PM_2.5_ and pneumonia and influenza (HR = 1.47, 95% CI= 1.27 to 1.71) was observed in a large representative cohort of US adults (13). Shorter-term exposure to PM_2.5_ air pollution has also been associated with elevated health-care visits for acute lower respiratory infection, including influenza in both children and adults (41).

This study has important strengths. It uses large cohorts of millions of well-defined and followed-up cancer patients and survivors. There is minimal risk of cohort selection bias, and the cohorts are representative of all regions tracked by SEER. The cohorts are large enough to stratify by specific cancer types. Air pollution and cohort data used in this analysis are publicly available at the county level.

This study also has notable limitations. As with all existing epidemiological studies of long-term exposure to air pollution, this study is unable to precisely assess lifetime exposure for individuals. Under the assumption of classical measurement error, it is sometimes assumed that ensuing measurement errors would result in biasing the PM_2.5_-mortality association toward the null. However, it is possible that PM_2.5_ exposure estimates are subject to Berkson error. Recent work showed that Berkson error in the presence of unmeasured confounding can result in biased estimates with the direction of the bias being unknown (42). Furthermore, there is potential of complex exposure measurement error that may not be strictly classical or Berkson in structure, with potential bias not fully understood (37).

Another limitation of this analysis is that it cannot fully account for potentially long and unknown latency periods of exposure and disease and that latency periods are likely different for different diseases. In this analysis, adverse associations for cardiopulmonary disease and PM_2.5_ were observed for alternative exposure windows and in temporally decomposed time-dependent analysis. Similar PM_2.5_-mortality associations across various exposure windows and high correlations in mean PM_2.5_ concentrations for different time periods make it difficult to make inferences regarding most relevant exposure windows. Another limitation of this study is the lack of individual-level controls for risk factors such as smoking, alcohol consumption, physical exercise, obesity, income, or education. This study used contextual variables to account for differences across counties, but there remains potential for residual confounding. Furthermore, the obesity and exercise data obtained from the Behavioral Risk Factor Surveillance System do not correct for reporting biases or correct reporting bias for the county-level body mass index means.

The objective of this study was to evaluate associations between PM_2.5_ exposure and risk for cancer, cardiovascular, and respiratory mortality in large cohorts of US cancer patients and survivors. The results provide evidence that PM_2.5_ exposure increases the risk of cardiovascular and respiratory mortality in a large vulnerable subpopulation of cancer patients and survivors. Given the pervasive and involuntary nature of air pollution exposure, and the large proportion of persons who will be diagnosed with cancer in their lifetime, these results are of substantial importance to public health and environmental policy.

## Funding

This work was supported by the Center for Air, Climate, and Energy Solutions, which was supported under Assistance Agreement (No. R835873) awarded by the US Environmental Protection Agency.

## Footnotes

**Role of funder:** The funder had no role in the study design; collection, analysis, and interpretation of the data; the writing of the manuscript; or the decision to submit the manuscript for publication.

**Disclaimer:** This work has not been formally reviewed by US Environmental Protection Agency (EPA). The views expressed in this document are solely those of authors and do not necessarily reflect those of the EPA. EPA does not endorse any products or commercial services mentioned in this publication.

**Disclosures:** ME reports a charitable grant from AstraZeneca Young Health Programme and personal fees from Prudential, Scor, and Third Bridge, all outside the submitted work. All other authors report no conflicts of interest.

**Author contributions:** Conceptualization: CAP, NCC, RTB; Data curation: NCC, CAP, JDM, ALR; Formal analysis: NCC, CAP, RTB; Methodology: NCC, CAP, RTB, ME, JDM; Supervision: CAP, ALR; Writing—original draft: NCC, CAP; Writing—review & editing: All authors have read and approved the final manuscript.

## Data Availability

Cancer incidence, county attributes, and related data are available from the US National Cancer Institute’s Surveillance, Epidemiology, and End Results Program (SEER). SEER data are accessible but require agreement to follow provisions that protect the identities of cancer patents and a signed SEER research data agreement (www.seer.cancer.gov). Air pollution estimates are available at the Center for Air, Climate, and Energy Solutions website (https://www.caces.us/).

## Supplementary Material

pkab001_Supplementary_Data
