# Supplementary material for: Fine Particulate Matter Air Pollution and Mortality Risk Among US Cancer Patients and Survivors
Source: JNCI Cancer Spectr. 2021 Feb 21;5(1):pkab001. doi: 10.1093/jncics/pkab001 (PMC7898081; doi:10.1093/jncics/pkab001)

SUPPLEMENTARY MATERIALS

Supplementary Table 1. The total number of cases and deaths for the primary cohort by diagnosed cancer type.

Diagnosed cancer type sub-cohorts	ICD 10 Codes	Total Cases	Total Deaths	Cause of Death						
				Cancer	Cardio-pulmona	Cardio-vascular	Cerebro-vascular	COPD	Pneumonia/ Influenza	Other
All Cancers	C00-97	5,591,168	2,273,354	1,686,958	324,394	204,028	41,734	59,328	19,304	262,002
Digestive Tract										
Oral	C00-14	130,941	57,426	42,778	8,181	4,916	1,033	1,598	634	6,467
Esophagus	C15	48,285	38,853	34,305	2,593	1,669	244	499	181	1,955
Stomach	C16	86,758	59,501	50,673	4,666	3,087	601	664	314	4,162
Small Intestine	C17	26,176	11,152	8,250	1,347	927	194	143	83	1,555
Colon	C18	341,271	167,920	114,567	29,829	19,650	3,967	4,532	1,680	23,524
Rectal	C19-21	179,072	74,250	53,837	11,109	7,305	1,370	1,837	597	9,304
Liver	C22-24	119,199	92,401	77,266	3,583	2,424	474	459	226	11,552
Pancreas	C25	124,203	107,798	102,009	2,716	1,841	429	295	151	3,073
Other Digestive	C26	6,198	5,294	4,786	207	148	15	32	12	301
Respiratory										
Nose	C30-31	9,274	4,458	3,493	520	328	80	86	26	445
Larynx	C32-33	41,143	21,622	14,587	4,478	2,485	450	1,266	277	2,557
Lung	C34	610,285	504,406	445,625	38,983	19,101	3,422	14,244	2,216	19,798
Other Respiratory	C37-39	16,095	10,588	9,367	631	413	61	112	45	590
Bone/Tissue										
Bone	C40-41	288,789	143,840	103,786	20,995	13,781	2,389	3,135	1,690	19,059
Skin	C43-44	407,673	71,614	36,811	18,770	12,481	2,743	2,474	1,072	16,033
Soft Tissue	C45-49	52,036	23,833	20,182	1,866	1,224	264	256	122	1,785
Sex-Specific										
Breast	C50	966,300	201,047	123,956	41,824	25,834	6,642	6,989	2,359	35,267
Other Female	C51-58	334,382	114,252	90,619	11,943	7,731	1,857	1,632	723	11,690
Prostate	C61	775,106	194,635	88,976	62,527	41,992	7,876	9,264	3,395	43,132
Other Male	C60,62	44,926	5,582	3,500	1,062	721	104	180	57	1,020
Urinary Tract										
Kidney	C64-65	158,962	58,650	40,080	9,837	6,593	1,277	1,390	577	8,733
Bladder	C67	186,021	78,487	43,564	21,198	13,299	2,336	4,490	1,073	13,725
Other Urinary	C66,68	9,320	4,997	3,445	909	582	95	181	51	643
Nervous System										
Brain	C71	86,146	52,713	48,474	1,805	1,038	463	168	136	2,434
Other Nervous	C69-	105,302	18,620	8,111	5,372	3,147	1,173	725	327	5,137
Other										
Endocrine	C73-75	190,923	16,730	9,573	3,677	2,366	622	455	234	3,480
Ill Defined	C76-80	246,382	132,685	104,338	13,766	8,945	1,553	2,222	1,046	14,581

* Cancer death is classified as C00-97; cardiopulmonary as I00-09, I11, I13, I20-51, I60-69, J40-47, J09-J18; cardiovascular as I00-09, I11, I13, I20-51; cerebrovascular as I60-69; COPD as J40-47; and Pneumonia and Influenza as J09-J18. Other was classified as all codes not cancer or cardiopulmonary.

Supplementary Table 2. Estimated hazard ratios (95% CIs) associated with 10 μg/m^3 increase of $PM_{2.5}$ and cancer and cardiopulmonary mortality using various exposure windows for the temporally decomposed analysis[a]

Model	Cohort Size	Cancer Deaths	Cardio-pulmonary Deaths	Cancer			Cardiopulmonary		
				1-Year Lag	6 year moving average	12 year moving average	1-Year Lag	6 year moving average	12 year moving average
2000	278,748	32,336	3,000	0.97 (0.90-1.05)	1.01 (0.93-1.09)	0.99 (0.93-1.07)	1.09 (0.86-1.39)	1.03 (0.80-1.32)	1.03 (0.83-1.29)
2001	528,223	63,798	6,238	0.99 (0.94-1.05)	0.98 (0.93-1.04)	0.97 (0.92-1.02)	1.28 (1.07-1.53)	1.32 (1.10-1.59)	1.29 (1.09-1.52)
2002	741,507	76,338	8,542	1.00 (0.96-1.05)	1.00 (0.95-1.06)	1.00 (0.95-1.05)	1.35 (1.18-1.56)	1.45 (1.23-1.72)	1.40 (1.20-1.63)
2003	934,036	83,432	10,386	0.92 (0.88-0.97)	0.94 (0.89-0.99)	0.94 (0.90-0.99)	1.09 (0.95-1.25)	1.11 (0.95-1.28)	1.07 (0.93-1.23)
2004	1,138,676	89,244	12,049	0.95 (0.90-1.00)	0.95 (0.90-1.00)	0.95 (0.90-0.99)	1.15 (0.99-1.32)	1.13 (0.99-1.30)	1.09 (0.96-1.24)
2005	1,335,118	94,746	13,988	0.99 (0.94-1.05)	1.00 (0.95-1.06)	1.00 (0.95-1.05)	1.15 (1.00-1.32)	1.17 (1.03-1.34)	1.13 (0.99-1.28)
2006	1,531,869	98,182	15,744	0.92 (0.87-0.98)	0.96 (0.91-1.01)	0.97 (0.92-1.02)	1.13 (0.96-1.32)	1.15 (1.00-1.32)	1.13 (1.00-1.29)
2007	1,740,678	102,510	17,186	0.94 (0.88-1.00)	0.96 (0.90-1.01)	0.97 (0.92-1.02)	1.25 (1.06-1.48)	1.22 (1.06-1.40)	1.20 (1.06-1.36)
2008	1,946,191	105,053	19,170	0.94 (0.89-0.99)	0.94 (0.89-1.00)	0.96 (0.91-1.01)	1.03 (0.91-1.17)	1.08 (0.94-1.24)	1.11 (0.98-1.25)
2009	2,148,227	108,746	21,286	0.92 (0.86-0.97)	0.92 (0.87-0.98)	0.96 (0.91-1.01)	1.34 (1.16-1.54)	1.41 (1.22-1.64)	1.37 (1.21-1.55)
2010	2,341,110	113,025	22,733	0.94 (0.87-1.01)	0.91 (0.86-0.98)	0.95 (0.90-1.00)	1.23 (1.05-1.44)	1.22 (1.05-1.41)	1.17 (1.04-1.32)
2011	2,530,627	113,666	24,557	0.90 (0.83-0.97)	0.91 (0.85-0.97)	0.94 (0.89-0.99)	1.27 (1.08-1.50)	1.28 (1.10-1.49)	1.20 (1.06-1.36)
2012	2,710,575	116,110	25,876	0.90 (0.84-0.97)	0.92 (0.86-0.99)	0.95 (0.89-1.01)	1.14 (0.97-1.33)	1.17 (1.00-1.36)	1.15 (1.02-1.31)
2013	2,882,730	118,835	28,024	0.93 (0.86-1.01)	0.92 (0.85-0.99)	0.94 (0.88-1.00)	1.33 (1.12-1.58)	1.23 (1.05-1.44)	1.20 (1.06-1.37)
2014	3,047,535	120,544	29,084	0.95 (0.89-1.02)	0.93 (0.87-1.01)	0.94 (0.88-1.01)	1.14 (1.00-1.31)	1.22 (1.04-1.43)	1.23 (1.07-1.42)
2015	3,192,710	122,493	31,562	0.95 (0.89-1.02)	0.94 (0.87-1.02)	0.98 (0.91-1.05)	1.23 (1.07-1.41)	1.24 (1.06-1.45)	1.23 (1.07-1.42)
2016	3,293,734	122,334	33,216	0.96 (0.88-1.04)	0.98 (0.91-1.06)	1.00 (0.93-1.07)	1.06 (0.90-1.24)	1.26 (1.08-1.47)	1.32 (1.15-1.51)
Random Effects Meta Estimate	--	--	--	0.95 (0.93-0.97)	0.95 (0.94-0.97)	0.96 (0.95-0.98)	1.19 (1.14-1.24)	1.21 (1.17-1.26)	1.19 (1.15-1.24)
Fixed Effect Meta Estimate	--	--	--	0.95 (0.94-0.96)	0.95 (0.94-0.97)	0.96 (0.95-0.98)	1.18 (1.14-1.23)	1.21 (1.17-1.26)	1.19 (1.15-1.23)

[a]Yearly Cohort estimates as well as the fixed and random effects results are included.

Supplementary Table 3. Estimated hazard ratios (95% CIs) associated with 10 µg/m^3 increase of $PM_{2.5}$ from 1999-2015 and cancer and cardiopulmonary mortality stratified for selected subgroups using the primary cohort.

Stratification	Cancer	Cardiopulmonary
All	0.99 (0.98-1.01)	1.25 (1.21-1.30)
Initially Diagnosed Cancer		
Oral	1.11 (1.00-1.23)	1.34 (1.07-1.69)
Esophagus	1.09 (0.97-1.22)	1.49 (0.97-2.30)
Stomach	1.03 (0.94-1.13)	1.59 (1.15-2.21)
Small Intestine	1.02 (0.81-1.29)	1.37 (0.74-2.51)
Colon	1.02 (0.96-1.09)	1.19 (1.04-1.35)
Rectal	1.12 (1.02-1.23)	1.12 (0.91-1.38)
Liver	1.01 (0.94-1.08)	1.57 (1.09-2.27)
Pancreas	1.00 (0.94-1.07)	1.22 (0.82-1.82)
Other Digestive	1.06 (0.73-1.52)	1.91 (0.23-15.70)
Nose	0.83 (0.58-1.19)	2.51 (0.82-7.69)
Larynx	1.07 (0.88-1.29)	1.29 (0.91-1.82)
Lung	0.98 (0.95-1.01)	1.27 (1.13-1.42)
Other Respiratory	1.00 (0.80-1.25)	3.33 (1.27-8.70)
Bone	1.01 (0.94-1.07)	1.40 (1.21-1.63)
Skin	1.29 (1.16-1.43)	1.37 (1.17-1.59)
Soft Tissue	0.87 (0.76-1.00)	1.38 (0.83-2.31)
Breast	1.09 (1.02-1.16)	1.25 (1.12-1.39)
Other Female	1.02 (0.95-1.10)	1.48 (1.21-1.82)
Prostate	0.98 (0.91-1.05)	1.15 (1.05-1.25)
Other Male	0.72 (0.52-1.01)	1.26 (0.64-2.47)
Kidney	0.94 (0.85-1.04)	1.12 (0.90-1.39)
Bladder	1.02 (0.92-1.13)	1.22 (1.06-1.42)
Other Urinary	1.12 (0.76-1.64)	1.50 (0.72-3.15)
Brain	0.93 (0.85-1.03)	1.76 (1.04-2.98)
Other Nervous	0.95 (0.75-1.20)	1.24 (0.92-1.68)
Endocrine	0.89 (0.72-1.11)	0.99 (0.70-1.42)
Ill Defined	1.06 (1.00-1.13)	1.49 (1.25-1.77)
Cancer Survivability*		
High Survival	1.05 (1.01-1.10)	1.20 (1.14-1.28)
Medium Survival	1.01 (0.99-1.04)	1.25 (1.18-1.33)
Low Survival	1.00 (0.98-1.03)	1.37 (1.27-1.49)
Any Chemo/Radiation		
None or Missing	0.98 (0.96-1.01)	1.21 (1.15-1.26)
Yes	1.06 (1.04-1.08)	1.33 (1.25-1.42)
Age Buckets		
0-18	0.92 (0.75-1.13)	1.42 (0.18-11.30)
19-39	1.01 (0.91-1.11)	0.74 (0.41-1.34)
40-54	0.98 (0.94-1.02)	1.32 (1.10-1.59)
55-65	0.98 (0.95-1.01)	1.21 (1.10-1.34)
65-85	0.99 (0.97-1.01)	1.26 (1.21-1.32)
Sex		
Male	0.99 (0.97-1.01)	1.24 (1.18-1.30)
Female	1.00 (0.98-1.03)	1.27 (1.19-1.35)
Race		
White	1.00 (0.98-1.01)	1.24 (1.19-1.29)
Black	0.92 (0.86-0.98)	1.42 (1.21-1.66)
Other	0.94 (0.87-1.02)	1.28 (1.01-1.60)
Stage		
In Situ (I)	1.03 (0.88-1.21)	1.35 (1.16-1.58)
Localized (II)	0.98 (0.94-1.02)	1.14 (1.07-1.22)
Regional (III)	0.97 (0.94-1.00)	1.31 (1.19-1.44)
Distant (IV)	0.98 (0.96-1.01)	1.45 (1.30-1.63)
Unknown or Missing	0.97 (0.94-1.01)	1.26 (1.18-1.34)

* The high survival cancers include skin, breast, prostate, other male, and endocrine cancers. The medium survival cancers include oral, small intestine, colon, rectal, nose, larynx, other respiratory, bone, soft tissue, other female, kidney, bladder, other urinary, and other nervous system. The low survival cancers include esophageal, stomach, liver, pancreatic, other digestive, lung, brain, and ill defined.

Supplementary Table 4. Sensitivity analysis for cancer and cardiopulmonary disease mortality using the primary cohort.

Sensitivity Analysis	Cancer	Cardiopulmonary
Covariate Selection [a]		
Only age, sex, race (in STRATA statement)	1.15 (1.14-1.15)	1.39 (1.37-1.41)
+ State, year, urban, marital status	1.09 (1.08-1.10)	1.33 (1.30-1.36)
+ Linear county variables	0.97 (0.96-0.99)	1.22 (1.19-1.26)
+ Quadratic county variables (Base Model)	0.99 (0.98-1.01)	1.25 (1.21-1.30)
+ Include Stage as Covariate	0.99 (0.97-1.01)	1.24 (1.20-1.29)
+ Include Stage in STRATA statement	0.99 (0.97-1.01)	1.25 (1.20-1.30)
Spatial Control [b]		
None	0.99 (0.98-1.00)	1.28 (1.25-1.32)
East versus West	1.09 (1.08-1.11)	1.37 (1.33-1.42)
Region	1.02 (1.00-1.03)	1.25 (1.20-1.29)
Registry	1.00 (0.99-1.02)	1.17 (1.12-1.22)
Cluster by County	0.99 (0.93-1.06)	1.25 (1.14-1.36)
Model Choice [c]		
1988-2015 Exposure Window	1.00 (0.99-1.02)	1.19 (1.16-1.23)
Age used as Time Axis	1.01 (1.00-1.03)	1.25 (1.20-1.30)
Fine-Gray Competing Risk	0.99 (0.98-1.01)	1.26 (1.21-1.30)

[a] Sensitivity analysis was performed with several specifications: 1) Only $PM_{2.5}$, age, sex, and race. 2) The first specification plus state, urban versus rural, and cohort year fixed effects. 3) The second specification plus linear terms for percent in the county that smoke, consume alcohol, are physically active, are obese, are uninsured, live in rural areas, below 150% poverty, unemployed, working class, did not graduate high school, graduated high school, and who did more school than high school as well as median income, home value, and rent. 4) The third specification plus quadratic terms for all of the linear covariates (Base Model). 5) The fourth specification plus stage as a covariate. 6) The fifth specification, but stage was included in the STRATA statement instead of as a covariate.

[b] Sensitivity to spatial controls were tested by removing the state indicator variables and running the same analysis using no spatial control, east versus west, region, or registry indicator variables. Additionally, a model that controlled for state using indicator variables while also clustering by county was estimated.

[c] Sensitivity analysis for the model selection was performed by estimating the following models: 1) Changing the $PM_{2.5}$ exposure window from 1999-2015 to 1988-2015. 2) Adding the year of cancer incidence to the STRATA statement in the PHREG procedure and using the age at death or the end of follow up as survival time. 3) A model that estimated hazard ratios using a Fine-Gray competing risks model to avoid bias from other causes of death.

Supplementary Figure 1. Illustration of the process to construct the temporally decomposed cohorts.

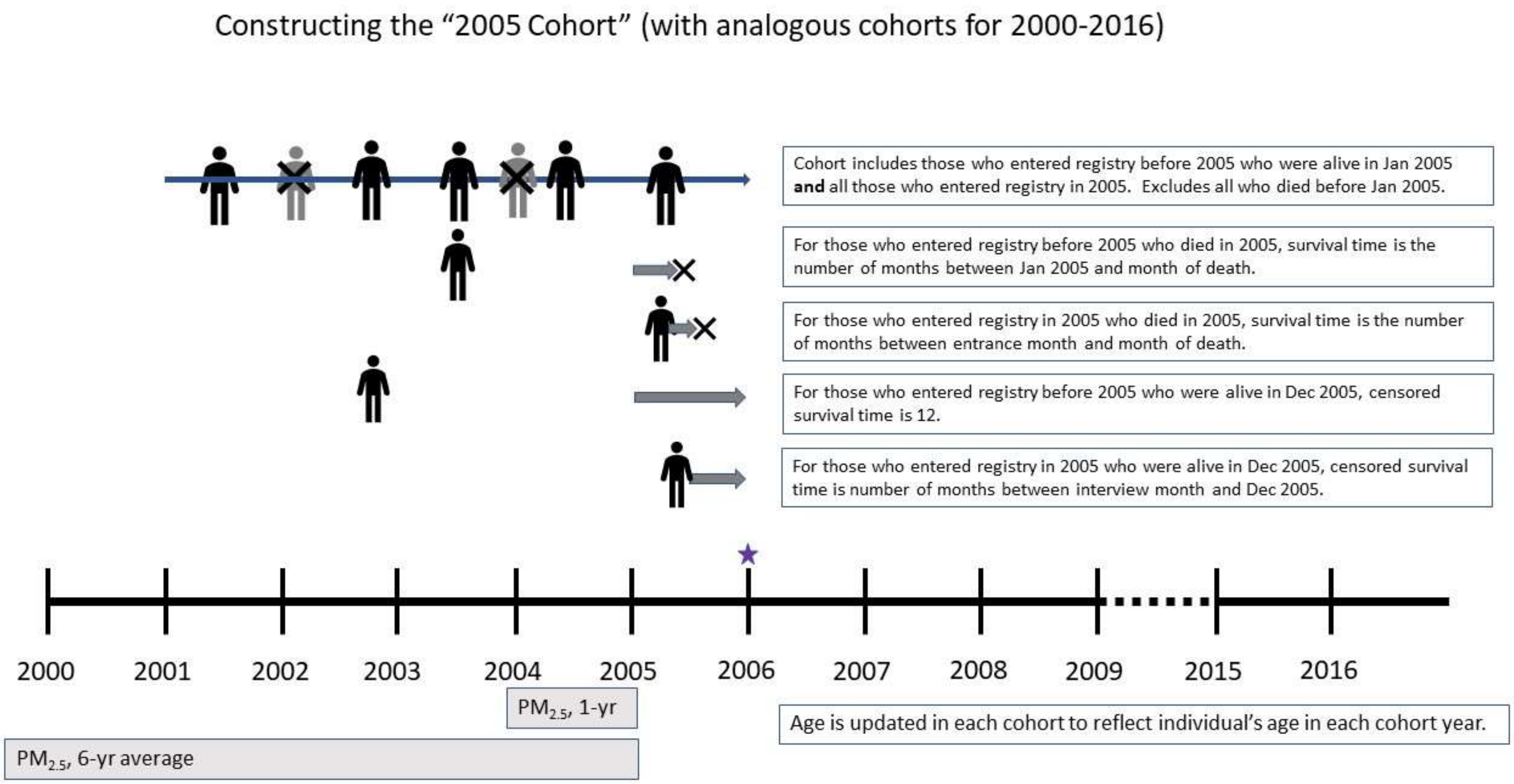

Supplement: pkab001_Supplementary_Data [file pkab001_supplementary_data.pdf]
